# Tailored, Therapist-Guided Internet-Based Cognitive Behavioral Therapy Compared to Care as Usual for Patients With Rheumatoid Arthritis: Economic Evaluation of a Randomized Controlled Trial

**DOI:** 10.2196/jmir.9997

**Published:** 2018-10-11

**Authors:** Maaike Ferwerda, Sylvia van Beugen, Henriët van Middendorp, Henk Visser, Harald Vonkeman, Marjonne Creemers, Piet van Riel, Wietske Kievit, Andrea Evers

**Affiliations:** 1 Health, Medical and Neuropsychology Department Institute of Psychology Leiden University Leiden Netherlands; 2 Medical Psychology Department Radboud University Medical Center Nijmegen Netherlands; 3 Department of Rheumatology Rijnstate Hospital Arnhem Netherlands; 4 University of Twente Enschede Netherlands; 5 Arthritis Center Twente Department of Rheumatology and Clinical Immunology Medisch Spectrum Twente Enschede Netherlands; 6 Department of Rheumatology Jeroen Bosch Hospital Den Bosch Netherlands; 7 Scientific Institute for Quality of Healthcare Radboud University Medical Center Nijmegen Netherlands; 8 Department for Health Evidence Radboud University Medical Center Nijmegen Netherlands

**Keywords:** cost-utility analysis, cognitive behavioral treatment, Internet-based therapy, rheumatoid arthritis

## Abstract

**Background:**

Internet-based cognitive behavioral therapy can aid patients with rheumatoid arthritis with elevated levels of distress to enhance their quality of life. However, implementation is currently lacking and there is little evidence available on the (cost-) effectiveness of different treatment strategies.

**Objective:**

Cost-benefit ratios are necessary for informing stakeholders and motivating them to implement effective treatment strategies for improving health-related quality of life (HRQoL) of patients with rheumatoid arthritis. A cost-effectiveness study from a societal perspective was conducted alongside a randomized controlled trial on a tailored, therapist-guided internet-based cognitive behavioral therapy (ICBT) intervention for patients with rheumatoid arthritis with elevated levels of distress as an addition to care as usual (CAU).

**Methods:**

Data were collected at baseline or preintervention, 6 months or postintervention, and every 3 months thereafter during the 1-year follow-up. Effects were measured in terms of quality-adjusted life years (QALYs) and costs from a societal perspective, including health care sector costs (health care use, medication, and intervention costs), patient travel costs for health care use, and costs associated with loss of labor.

**Results:**

The intervention improved the quality of life compared with only CAU (Δ QALYs=0.059), but at a higher cost (Δ=€4211). However, this increased cost substantially reduced when medication costs were left out of the equation (Δ=€1863). Of all, 93% (930/1000) of the simulated incremental cost-effectiveness ratios were in the north-east quadrant, indicating a high probability that the intervention was effective in improving HRQoL, but at a greater monetary cost for society compared with only CAU.

**Conclusions:**

A tailored and guided ICBT intervention as an addition to CAU for patients with rheumatoid arthritis with elevated levels of distress was effective in improving quality of life. Consequently, implementation of ICBT into standard health care for patients with rheumatoid arthritis is recommended. However, further studies on cost reductions in this population are warranted.

**Trial Registration:**

Nederlands Trial Register NTR2100; http://www.trialregister.nl/trialreg/admin/rctview.asp?TC=2100 (Archived by WebCite at http://www.webcitation.org/724t9pvr2)

## Introduction

The psychological impact of rheumatoid arthritis has become increasingly apparent, with patients reporting decreased health-related quality of life (HRQoL) as a result of physical factors, such as pain, and psychological factors, such as negative mood [1-3]. As these factors are associated with the disease trajectory, health care utilization, and workplace disability of patients [4-8], they often lead to significant societal health expenses [9-12].

Approximately one-third patients with rheumatoid arthritis experience a significantly reduced HRQoL [1,3], and cognitive behavioral therapy can aid in improving HRQoL [13-15]. In a recent randomized controlled trial (RCT), we demonstrated that a therapist-guided internet-based cognitive behavioral therapy intervention (ICBT) that was tailored to specific problems of individual patients with rheumatoid arthritis with elevated levels of distress led to improvements in their psychological functioning (eg, depressed mood) [16]. Findings of this study were in agreement with those of previous studies on face-to-face cognitive behavioral therapies [17], which our treatment protocol closely resembled. Furthermore, it also supported previous preliminary evidence suggesting that ICBT is as effective as face-to-face treatments for a range of somatic conditions and symptoms [18-20].

Several benefits of internet-based treatments, including increased flexibility in terms of time and place, may make these treatments feasible for widespread implementation [21,22]. Although evidence on cost-effectiveness of internet-based therapy is still scarce, preliminary results suggest that such interventions are a cost-effective method to improve mental health, specifically when guidance is provided by a psychological therapist [23]. One study examining a self-management intervention for patients with RA reported a reduction in general distress and pain, and improvement in self-efficacy, although no effects on health care utilization were seen [24], which is important for evaluation of costs. Furthermore, improved quality of life in patients with chronic somatic conditions has previously been associated with an improvement in medication adherence, self-efficacy, and positive health outcomes [10,12]. This could potentially reduce the cost of health care through, for example, greater adherence to medications and increased employability and work outcomes. However, the evidence on this is still scarce.

Specifically, no studies have examined the cost-effectiveness of ICBT in patients with rheumatoid arthritis thus far. This information is essential for allowing stakeholders to balance treatment choices and policy decisions. For example, a recent study summarized how rheumatologists balanced multiple aspects of a treatment choice, including efficacy, patient preferences, and costs [25].

This study describes a preplanned cost-effectiveness study conducted from a societal perspective on the use of a tailored, therapist-guided ICBT protocol as an addition to care as usual (CAU) for patients with rheumatoid arthritis with elevated levels of distress. This was conducted alongside RCT, the results of which have been reported elsewhere [16]. We predicted that ICBT would be a cost-effective intervention as addition to CAU. In particular, costs that are relevant to society, that is, health care sector costs such as medication costs, health care usage costs, and work-related costs, were examined as these potentially decrease with improving HRQoL.

## Methods

### Design

An economic evaluation of a tailored, therapist-guided ICBT protocol as an addition to CAU was conducted from a societal perspective alongside an RCT. Patients with elevated levels of distress were randomly selected to receive standard rheumatological care (as usually conducted in the Netherlands) only or in combination with ICBT. Further details of the RCT can be found in a previous study reporting the effects of ICBT on the psychological functioning, physical functioning, and impact of rheumatoid arthritis on daily life [16]. This study focuses only on aspects relevant to economic evaluation. All patients provided written informed consent for participation in the study. The regional medical ethical committee approved the study (NL24343.091.08), and it was registered with the Nederlands Trial Register (NTR2100).

### Participants

Adult patients with a rheumatologist-certified diagnosis of rheumatoid arthritis [26] and receiving out-patient standard rheumatological care at 1 academic and 3 nonacademic hospitals were invited to participate in this study. Only patients with elevated levels of distress, as defined by high scores for negative mood (≥21 for negative mood on the Impact of Rheumatic Diseases on General Health and Lifestyle scale) [27] and/or anxiety (a score of ≥5 for anxiety on Impact of Rheumatic Diseases on General Health and Lifestyle scale) were included. The exclusion criteria were (1) insufficient command of the Dutch language, (2) severe physical or psychiatric comorbidity (ie, requiring acute or intensive medical attention; when this was not the case, patients indicated which condition impacted their HRQoL to a greater extent), (3) pregnancy, (4) currently receiving treatment from a cognitive behavioral therapist or comparable practitioner, and (5) no access to a computer and internet.

### Care as Usual and Internet-Based Cognitive Behavioral Intervention

CAU for patients with rheumatoid arthritis, which was provided to the intervention and control groups, generally consists of shared care checkups provided every 3-6 months by a rheumatology nurse and the rheumatologist to monitor disease activity and treatment. Hospitals in the Netherlands follow the recommendations for rheumatological care provided by the Dutch Society for Rheumatology. In addition, physical and occupational therapy may be provided, depending on the patient and disease characteristics.

The intervention group received ICBT as an addition to CAU. The treatment was tailored to individual patient goals and characteristics and guided by a therapist. The treatment commenced with 1 or 2 face-to-face intake sessions comprising formulation of individual goals based on the main problems of the patient. Based on these goals, specific treatment modules embedded within the ICBT website were selected, and the therapist guided the choice of assignments within each of these modules based on the specific risk and resilience factors of the patient. Therapists and patients remained in contact weekly or biweekly (based on patient preferences) via a secured messaging service within the ICBT website. Treatment modules focused on coping with (1) pain and functional disability, (2) fatigue, (3) social functioning, and (4) negative mood. As the modules were tailored to individual requirements, treatment durations varied from 9 to 65 (mean 26.07, SD 12.22) weeks. All 6 therapists had a master’s degree in psychology and 2 had additional postacademic training in cognitive behavioral therapy. Supervision was provided by a senior clinical psychologist with postacademic training in cognitive behavioral therapy. Patients received 1 telephonic session that lasted for 30 minutes, where a research assistant explained how the intervention website was set up. Further information on the ICBT intervention can be found in our previous study [16].

### Data Collection and Outcome Measures

Data were collected at baseline; postintervention for the intervention group and 6 months after baseline for the control group; and at 3 (F1), 6 (F2), 9 (F3), and 12 months (F4) thereafter. All questionnaires were filled out in paper and pencil versions. All costs were calculated based on the 2015 Dutch price indices. The last observation carried forward was applied for missing data to account for biases introduced by nonresponse.

#### Effects: Quality-Adjusted Life Years

HRQoL was assessed using the Dutch version of the EuroQol-5dimensions-3levels (EQ-5D-3L) questionnaire [28]. EQ-5D-3L captures 5 dimensions of health, including mobility, self-care, usual activities, pain or discomfort, and anxiety or depression. Each dimension has 3 response options: no, some or moderate, and extreme problems. Utility scores were calculated using the Dutch tariff [28], with scores ranging from 0 (death) to 1 (perfect health). The trapezium rule was applied for calculating area under the curve for measuring quality-adjusted life years (QALYs).

#### Costs: Societal Perspective

Costs were calculated for 3 dimensions, including health care sector costs (comprising health care use, medication, and costs of the intervention under study), patient travel costs for health care use, and costs associated with loss of labor (absenteeism and presenteeism).

Health care use was assessed using the Trimbos or Institute for Medical Technology Assessment questionnaire for Costs associated with Psychiatric Illness (TiC-P) [29], which was adjusted for health care use by patients with rheumatoid arthritis. The questionnaire included patient appointments with rheumatologists, specialized rheumatology nurses, occupational therapists, physical therapists, podiatrists, and hydrotherapists; admissions to daycare; and inpatient treatment at hospitals or rehabilitation centers. Furthermore, TiC-P also assessed care provided by a general practitioner and occupational health doctor, psychological or psychosocial care (eg, care provided by a psychologist, psychiatrist, or social worker), and care provided by alternative medicine practitioners. Costs were calculated by multiplying health care use with estimates of unit prices, as provided by the TiC-P [29] and the Dutch manual for cost analyses in health care [30].

All medications related to rheumatoid arthritis were taken into account, including pain medication (eg, nonsteroidal antiinflammatory drugs), corticosteroids, disease modifying antirheumatic drugs, and biologics. Furthermore, medications related to psychological symptoms, such as depression and anxiety, as well as those related to sleep disorders were also taken into consideration. Costs of these medications were calculated by multiplying dosages for each type of medication with their costs as per the Dutch national tariff list. The medication history was self-reported by the patients.

In accordance with the Dutch guidelines for cost analyses in health care [30], costs for the tailored, therapist-guided ICBT protocol were calculated using (1) the actual costs of development of the intervention by the ICT-company, which included updating and security costs, (2) salary of the therapists (as per rates for basic psychologists and those with a postdoc training, where appropriate) based on the amount of time they spent on treatment for each patient (including face-to-face intake sessions, internet-based communication, and additional telephone calls), (3) salary costs for the research assistant conducting the telephone session to explain the intervention website, and (4) patient traveling expenses for the face-to-face intake sessions. An amortization period of 5 years was assumed. Costs per patient were calculated based on prevalence rates of rheumatoid arthritis provided by the Netherlands Institute for Health Services Research [31]. An assumption was made that 30% of the rheumatoid arthritis population is eligible for this intervention because of elevated levels of distress, as observed in the RCT [16] and a previous trial targeting the same population [17]. Of this population size, a population reach of 10% was assumed.

Patient travel costs were calculated by multiplying the Dutch standard for average travel distances from home to several health care services (for example, hospital, general practitioner, and physical therapist), as per the Dutch manual for cost analysis in health care [30], with a price of €0.19 per kilometer.

Loss of productivity costs were calculated for 1 year using the friction costs method including presenteeism and absenteeism, based on self-reported data on loss of productivity collected via the PROductivity and DIsease Questionnaire (PRODISQ) [32]. The friction period was calculated to a maximum of 12 weeks [30]. An additional period of 4 weeks was taken into consideration to allow management to fill the vacancy. Loss of productivity costs was calculated by multiplying overall average costs of productivity loss per hour (€34.90) [30] with the number of hours that a patient was absent from work or was unable to perform optimally at work because of rheumatoid arthritis.

### Statistical Analysis

Differences in baseline sociodemographic (eg, age and gender), disease-related (eg, disease severity), and economic (eg, paid labor, health care costs, medication costs, and HRQoL) characteristics between the intervention and control groups were assessed using independent sample *t* tests or chi-square tests as appropriate.

The costs per QALY gained were assessed using an incremental cost-utility ratio (ICUR), calculated by dividing the difference in costs by the difference in QALYs. Bootstrapping (1000 replications) was used to nonparametrically determine 95% CI. Results of the bootstrap were presented and analyzed by means of a cost-effectiveness plane and willingness-to-pay curve. The Dutch Council for Public and Health Care (RVZ) recommends that the threshold of the ICUR in relation to the acceptability of the treatment has to depend on the severity of the disease with a maximum ICUR of €80,000/QALY [33]. In accordance, the probability that this intervention remains within this threshold for willingness to pay is reported. As the intervention was not primarily aimed at reducing medication costs and a substantial proportion of patients with rheumatoid arthritis used expensive biologic agents that strongly influenced cost estimations, a secondary analysis was performed where costs for medication were excluded.

## Results

### Patient Demographics

Patient demographics have been shown in Table 1 and Table 2. This study included 133 patients, of which 62 patients were in the ICBT group and 71 were in the CAU group. Measurements at all time-points were provided by 27% (17/62) patients allocated to the ICBT group and 42% (30/71) patients allocated to the CAU group. No baseline differences in demographics, disease-related characteristics, and cost- or effect-related variables were observed. The patient sample, which included more female than male patients (85 female and 48 male) and had a mean age of 56.35 (SD 10.00; range: 26-81) years, was a representative of patients with rheumatoid arthritis.

### Between-Group Differences in Effects: Quality-Adjusted Life Years

An overview of EQ-5D-3L utility scores for the intervention and control groups has been shown in Table 3, based on a 1-year follow-up period, indexed to the year 2015, for primary and secondary analyses. Although HRQoL was found to be similar for the intervention and control groups at baseline (*P*=.16), the former exhibited a higher QALY score (mean QALY=0.86, 2.5-97.5 percentile=0.82-0.89) than the latter (mean QALY=0.80, 2.5-97.5 percentile=0.76-0.83) during the 1-year follow-up period.

### Between-Group Differences in Costs: Societal Perspective

The intervention and control groups did not exhibit any differences with regard to all costs during the 1-year follow-up period (Table 4), based on a 1-year follow-up period, indexed to the year 2015. Total costs for the intervention amounted to €419 per patient.

**Table 1:** Baseline patient characteristics: continuous variables.

Group	Characteristics			
	EQ-5D-3L^a^		Disease activity (RADAI^b^)	
	Mean (SD)	Range	Mean (SD)	Range
CAU^c^ (N=71)	0.69 (0.23)	−0.11 to 1.0	3.84 (1.75)	0.40 to 7.27
ICBT^d^ + CAU (N=62)	0.74 (0.19)	0.09 to 1.0	3.31 (1.99)	0.20 to 7.95
Total group (N=133)	0.71 (0.21)	−0.11 to 1.0	3.59 (1.88)	0.20 to 7.95

^a^EQ-5D-3L: EuroQol-5dimensions-3 levels. Outcome analysis; group differences at baseline were analyzed using independent samples *t* tests as appropriate. *P*=.16.

^b^RADAI: Rheumatoid Arthritis Disease Activity Index. Group differences at baseline were analysed using independent samples *t* tests as appropriate. *P*=.11.

^c^CAU: care as usual.

^d^ICBT: internet-based cognitive behavioral therapy.

**Table 2:** Baseline patient characteristics: dichotomous variables.

Characteristics		CAU^a^ (N=71)			ICBT^b^ + CAU (N=62)			Total group (N=133)			*P* value^c^
		Yes	No	Missing	Yes	No	Missing	Yes	No	Missing	
Medical comorbidity		36	32	3	30	30	2	66	62	5	.74
Psychological comorbidity		5	63	3	2	58	2	7	121	5	.32
Employed		28	40	3	28	33	1	56	73	4	.59
**Medication use**											
	Painkillers	8	56	7	10	46	6	18	102	13	.41
	NSAIDS^d^	29	35	7	23	33	6	52	68	13	.64
	DMARDS^e^	52	12	7	46	10	6	98	22	13	.90
	Corticoids	13	51	7	12	44	6	25	95	13	.88
	Biologicals	21	43	7	24	32	6	45	75	13	.26
	Mental Health Medication	6	58	7	4	52	6	10	110	13	.66

^a^CAU: care as usual.

^b^ICBT: internet-based cognitive behavioral therapy.

^c^Outcome analysis; group differences at baseline were analyzed using chi-square analysis or independent samples *t* tests as appropriate.

^d^NSAID: Nonsteroidal antiinflammatory drugs.

^e^DMARD: disease modifying antirheumatic drugs.

**Table 3:** Quality-adjusted life years and costs for the care as usual and internet-based cognitive behavioral therapy plus care as usual groups.

Analysis		CAU^a^		ICBT^b^ + CAU		Δ QALY^c^	Δ Costs
		QALY	Costs	QALY	Costs		
**Primary analysis**							
	Average	0.80	€11,542	0.86	€15,754	0.059	€4211
	2.5 percentile	0.76	€11,830	0.82	€8671	0.007	−€636
	97.5 percentile	0.83	€20,134	0.89	€14,599	0.090	€9481
**Secondary analysis, excluding medication costs**							
	Average	0.80	€2846	0.86	€4774	0.0590	€1863
	2.5 percentile	0.76	€1743	0.82	€2541	0.007	−€714
	97.5 percentile	0.83	€4243	0.89	€7777	0.090	€5428

^a^CAU: care as usual.

^b^ICBT: internet-based cognitive behavioral therapy.

^c^QALY: quality-adjusted life years.

**Table 4:** Mean costs for the care as usual and the internet-based cognitive behavioral therapy plus care as usual groups.

Cost category	CAU^a^, mean (SD)	ICBT^b^ + CAU, mean (SD)	Difference between groups *P* value
Health care use	€2548 (3659)	€3252 (8477)	.52
Medication use	€8682 (12,469)	€10,901 (13,257)	.32
Patient travel costs	€109 (135)	€151 (160)	.93
Absenteeism	€363 (1258)	€1309 (9106)	.89
Presenteeism	€1800 (5853)	€2239 (7133)	.91
ICBT intervention	N/A^c^	€419	N/A

^a^CAU: care as usual.

^b^ICBT: internet-based cognitive behavioral therapy.

^c^N/A: not applicable.

**Figure 1:**
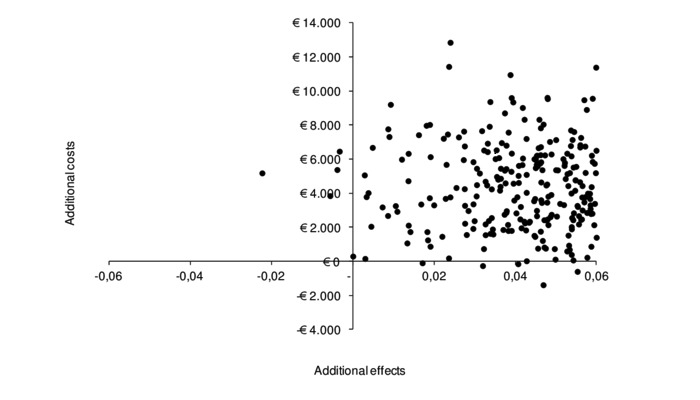
The cost-effectiveness plane of simulated incremental cost-effectiveness ratios of internet-based cognitive behavioral therapy as an addition to care as usual.

**Figure 2:**
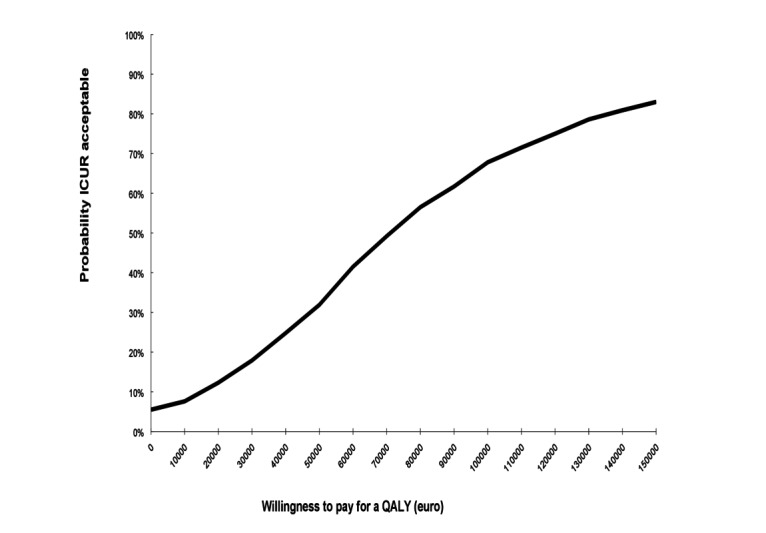
Cost-effectiveness acceptability curve comparing internet-based cognitive behavioral therapy plus care as usual group to care as usual alone group. ICUR: incremental cost-utility ratio; QALY: quality-adjusted life year.

Results of the cost-utility analysis have been presented in Table 3. Incremental effectiveness resulted in an effect of 0.059, whereas incremental costs amounted to €4211.44 (2.5-97.5 percentile=−€636-9481). Therefore, the incremental cost- effectiveness amounted to an investment of €71,424.82 costs per QALY gained. The cost-effectiveness plane containing a scatterplot of simulated ICURs has been shown in Figure 1. Majority (93%, 930/1000) of simulated ICURs were in the north-east quadrant, indicating a high probability that the intervention was effective in improving HRQoL, but at a greater cost for society compared with CAU. A total of 6% (60/1000) of ICURs were in the south-east quadrant, suggesting greater HRQoL effects at lower costs to society, and 1% (10/1000) of ICURs were in the north-west quadrant, suggesting lower HRQoL effects at higher costs to society (Figure 1). At a willingness to pay of €80,000, the intervention had a 57% chance of being cost-effective (Figure 2) for patients with rheumatoid arthritis with elevated levels of distress.

**Figure 3:**
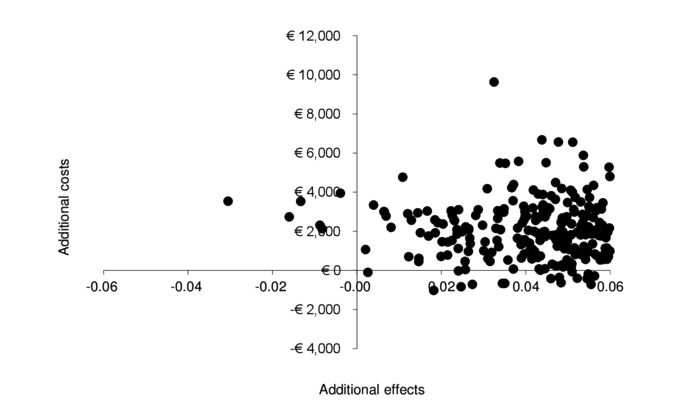
Secondary analysis excluding medication costs: Cost-effectiveness plane of simulated incremental cost-effectiveness ratios of internet-based cognitive behavioral therapy as an addition to care as usual.

**Figure 4:**
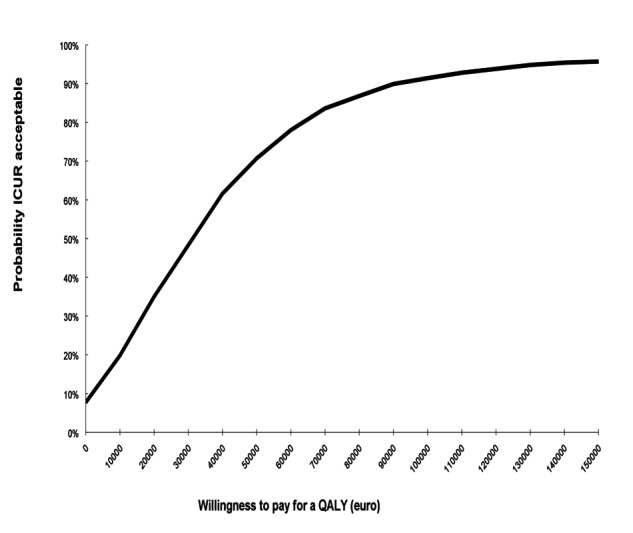
Secondary analysis excluding medication costs: Cost-effectiveness acceptability curve comparing the internet-based cognitive behavioral therapy plus care as usual group to care as usual alone group.

Upon repeating the analysis without taking the medication costs into account, incremental costs were seen to reduce to €1862.72 (2.5-97.5 percentile=−€714-€5428). The scatterplot of the simulated ICURs remained approximately the same (Figure 3), excluding costs of medication; however, the cost-effectiveness acceptability curve exhibited an 87% chance of being cost-effective at a willingness to pay of €80,000 (Figure 4) for patients with rheumatoid arthritis with elevated levels of distress, without taking medication costs into account.

## Discussion

This study was conducted with the aim of gaining insight into costs and effects of a tailored, therapist-guided ICBT protocol as an addition to CAU for patients with rheumatoid arthritis and elevated levels of distress. The key findings were (1) a positive effect on QALYs was observed in the intervention group compared with that in the control group; (2) cost ratios showed that this effect came at a greater cost to society; and (3) substantial costs in this population were generated by medications, and there were no group differences with regard to this. The cost-benefit ratio improved when costs of the medications were excluded. Based on effects of the intervention on improving the quality of life, implementation of the intervention is recommended; however, with respect to its effect on the costs, further study is warranted.

Results of this study are in agreement with those of previous studies that reported promising results with regard to the cost-effectiveness of therapist-guided psychological interventions [22,23]. Guidance by a therapist comes at a cost, which accumulates with the duration of treatment. Reducing therapist time by, for example, using more automated or prewritten responses could be beneficial in terms of costs, but comes with the possible risk of losing tailored aspects of the intervention. Future research should compare the cost-effectiveness of a guided, partially-guided, or nonguided intervention to identify the optimal amount of guidance necessary for obtaining cost-effective results. Total costs of the intervention per patient were very low in comparison to the other costs accounted for, which makes the intervention a relatively cheap addition to standard care for patients who might benefit from this intervention in terms of their HRQoL.

Medication costs within the field of rheumatoid arthritis have received considerable attention as biologics have a relatively high cost, and a similar finding was observed in this study. Although in the past there have been some indications that improved psychological functioning increases medication adherence and lowers medication use in the long term [12], no group differences in medication costs were observed in this study. Exclusion of medication costs from the analysis showed a more beneficial cost-effectiveness ratio of the ICBT intervention. Although adherence to medication was not an explicit goal of this intervention, it would be worthwhile to examine the ability of internet-based interventions in changing medication adherence patterns and medication use in patients with rheumatoid arthritis [34]. These interventions could include, for example, motivational interviews aimed at adherence [35]. Additional societal gains could also be attained by finding ways to enable patients in actively participating in the workforce [36,37].

The results of this study should be interpreted with caution because of the presence of missing data, because not all patients filled out all required measurements for the economic evaluation. Although the last observation carried forward method was applied for missing values, missing values can potentially lead to biases in results.

In conclusion, the tailored, therapist-guided ICBT intervention in patients with rheumatoid arthritis with elevated levels of distress rendered higher effects on HRQoL with higher costs. However, society may be willing to pay for the intervention as these costs remain within the threshold generally stated for health care interventions in the Netherlands. The findings of this study were in support of implementation of the intervention as a potential addition to CAU for patients with rheumatoid arthritis with elevated levels of distress, although future studies are necessary for optimizing the cost-benefit ratio.

## Abbreviations

CAU: care as usual
EQ-5D-3L: EuroQol-5dimensions-3levels
HRQoL: health-related quality of life
ICBT: internet-based cognitive behavioral therapy
ICUR: incremental cost-utility ratio
QALY: quality-adjusted life year
RCT: randomized controlled trial
TiC-P: Costs associated with Psychiatric Illness
